# Oxytocin and Cortisol Levels in Dog Owners and Their Dogs Are Associated with Behavioral Patterns: An Exploratory Study

**DOI:** 10.3389/fpsyg.2017.01796

**Published:** 2017-10-13

**Authors:** Maria Petersson, Kerstin Uvnäs-Moberg, Anne Nilsson, Lise-Lotte Gustafson, Eva Hydbring-Sandberg, Linda Handlin

**Affiliations:** ^1^Endocrine and Diabetes Unit, Department of Molecular Medicine and Surgery, Karolinska Institutet, Stockholm, Sweden; ^2^Department of Animal Environment and Health, Swedish University of Agricultural Sciences, Skara, Sweden; ^3^Department of Anatomy, Physiology and Biochemistry, Swedish University of Agricultural Sciences, Uppsala, Sweden; ^4^School of Health and Education, University of Skövde, Skövde, Sweden

**Keywords:** oxytocin, cortisol, dog–human interaction, behavior

## Abstract

We have previously shown that dog–owner interaction results in increasing oxytocin levels in owners and dogs, decreasing cortisol levels in owners but increasing cortisol levels in dogs. The present study aimed to further investigate whether oxytocin and cortisol levels in the previously tested owners and dogs were associated with their behaviors during the interaction experiment. Ten female volunteer dog–owners and their male Labrador dogs participated in a 60 min interaction experiment with interaction taking place during 0–3 min and blood samples for analysis of oxytocin and cortisol were collected at 0, 1, 3, 5, 15, 30, and 60 min. The entire experiment was videotaped and the following variables were noted; the different types (stroking, scratching, patting and activating touch, i.e., scratching and patting combined) as well as the frequency of touch applied by the owner, the number of times the owner touched her dog, the dog’s positions and time spent in each position. Correlations were analyzed between the behavioral variables and basal oxytocin levels, maximum oxytocin levels, delta oxytocin levels, basal cortisol levels and cortisol levels at 15 min. Owners with low oxytocin levels before and during the interaction touched their dogs more frequently (0 min: *R*_s_ = -0.683, *p* = 0.042; oxytocin maximum: *R*_s_ = -0.783, *p* = 0.013). The lower the dogs’ oxytocin levels during the interaction, the more stroking they received (*R*_s_ = -0.717, *p* = 0.041). The more frequently activating touch was applied by the owner, the higher the dogs’ cortisol levels became (15 min: *R*_s_ = 0.661, *p* = 0.038). The higher the owners’ maximum oxytocin level the fewer position changes the dogs made (*R*_s_ = -0.817, *p* = 0.007) and the shorter time they spent sitting (*R*_s_ = -0.786, *p* = 0.036), whereas the higher the owners’ basal cortisol levels, the longer time the dogs spent standing (0 min: *R*_s_ = 0.683, *p* = 0.041). In conclusion, oxytocin and cortisol levels, both in dogs and in their owners, are associated with the way the owners interact with their dogs and also with behaviors caused by the interaction.

## Introduction

In some human societies dogs have become a central part to family life and can even be considered as family members (Walsh, 2009a,b). The attachment relationship between a dog owner and its dog can be regarded as functionally similar to that seen between a parent and child (Topál et al., 1998; Palmer and Custance, 2008). Several studies have demonstrated that this type of relationship shows behavioral and neuroendocrine similarities to that described for mothers and infants (Serpell, 2004; Stoeckel et al., 2014; Nagasawa et al., 2015).

The neuropeptide oxytocin is produced in the paraventricular and supraoptic nuclei in the hypothalamus and is known to stimulate milk ejection during breastfeeding and uterine contractions during labor (Burbach et al., 2006). However, oxytocin is not only released during labor and breastfeeding, but may also be released by non-noxious sensory stimulation such as gentle touch. Both animals and humans respond to this type of stimulation, which induces, for example, anti-stress effects (e.g., decreased cortisol levels and blood pressure) (Uvnäs-Moberg, 1998), increased function of the gastrointestinal tract (Petersson et al., 1999), as well as increased pain threshold (Petersson et al., 1996).

In addition, non-noxious sensory stimulation in the context of intraspecies friendly social interaction, as for example in pair bonding, maternal behavior and attachment, is associated with activation of the oxytocinergic system (e.g., Carter, 1998; Uvnäs-Moberg et al., 2005). Oxytocin also facilitates bonding between mothers and young (e.g., the prairie vole: Carter, 1998; Insel et al., 1998; Sheep: Keverne and Kendrick, 1994; Humans: Uvnäs-Moberg, 1996; Feldman et al., 2007).

Studies in humans have shown that oxytocin, when applied through nasal spray, can stimulate certain aspects of social interactions, such as increase the ability to interpret tone of voice (Hollander et al., 2007) and facial expression (Domes et al., 2007a) and facilitate friendly social interactions (Domes et al., 2007b). It also increases trust (Kosfeld et al., 2005) and causes anti-stress and anxiolytic effects (Heinrichs et al., 2003). In similar ways, it has been shown that high endogenous oxytocin levels in mothers is related to the mothers being more interactive with their children, less anxious and more sensitive to their children’s cues (Uvnäs-Moberg et al., 1990; Feldman et al., 2007).

Also interaction between humans and dogs, which include pleasant non-noxious sensory stimulation, can induce oxytocin release in both humans and dogs and generate effects such as decreased cortisol levels and blood pressure (Odendaal and Meintjes, 2003; Miller et al., 2009; Handlin et al., 2011). In addition, dogs have been shown to be able to interpret their owner’s cues in different situations (Miklósi, 2009). Recent research has indicated that oxytocin can influence the social behavior of dogs toward humans, for example polymorphisms in the oxytocin receptor gene in dogs have been associated with differences in human directed behavior (Kis et al., 2014), nasally administered oxytocin increased gazing behaviors in dogs (Nagasawa et al., 2015) and oxytocin enhanced performance using momentary distal pointing cues (Oliva et al., 2015).

We have previously shown that interaction between dog owners and their dogs results in increasing levels of oxytocin in both owners and dogs, whereas cortisol levels decrease in the owners but increase in the dogs (Handlin et al., 2011). In addition, higher oxytocin levels in both owners and dogs, and lower levels of cortisol in the owners, are related to the owner’s description of the owner-dog relationship as being pleasant and interactive and associated with fewer problems. We could also show that the owners’ and the dogs’ oxytocin levels are closely related (Handlin et al., 2012). Based on our previous results we expected that touch and behaviors related to calm and anti-stress would be positively related to oxytocin levels in both dogs and owners, whereas behaviors related to activation or stress would be associated with cortisol levels. The overall aim of the study was therefore to investigate whether oxytocin and cortisol levels in the previously tested owners and dogs were associated with their behaviors and more specifically we wanted to address the following questions: (1) Is the frequency and type of touch initiated by the owner associated with oxytocin levels in owners and dogs? (2) Is the frequency and type of touch initiated by the owner associated with cortisol levels in owners and dogs? (3) Are owners’ oxytocin and cortisol levels associated with the dogs’ behavior?

## Materials and Methods

The results of the present paper is based on data described in detail in previous manuscripts (see Handlin et al., 2011, 2012). Those parts of materials and methods, which are of relevance for the present paper will be summarized below.

### Setting and Participants

Ten privately owned male Labrador retrievers, older than 1 year (mean age = 4.7 years; *SD* = 3 years) and their female middle-aged owners (mean age = 53 years; *SD* = 10 years), with whom they had been living together with during their entire lives, were recruited to the study by information given at veterinarian clinics and local workplaces. The owners were informed that the overall aim of the study was to investigate positive consequences of the human-dog relationship. The owners were to take part in an interaction experiment during which both owners and dogs would be exposed to blood sampling for analysis of hormones, such as oxytocin and cortisol. In addition the experiment would be videotaped for behavioral analysis. The owners were also informed about some additional measurements (such as heart rate etc.) that are not described in the present paper.

The study was conducted in an ordinary room (∼4 by 5 m) with four chairs, a desk and a bookcase. Dogs had *ad libitum* access to water during the testing. The experiments were performed in either the mornings or the evenings, depending on the participants’ work schedules. The study was conducted at the Swedish University of Agricultural Sciences in Skara, Sweden.

### Interaction Experiment

The owner was sitting in a chair with her dog lying or sitting beside her before the start of the experiment. At time-point zero the owner approached her dog and started to interact with him by talking to the dog and by touching different parts of the dog’s body for 3 min. The owner was instructed to interact with her dog in the same way as they usually do at home. After the 3 min of interaction the owner was instructed not to touch or talk to the dog for the rest of the experiment and to remain seated in her chair. The whole experiment lasted for 60 min. However, in most cases, the owner occasionally touched and talked to her dog in order to correct his behavior during the remaining part of the experiment.

### Blood Sampling and Hormone Analysis

As previously described in Handlin et al. (2011), an indwelling catheter was inserted into the cubital vein of the dog owners and an intravenous catheter was inserted into the cephalic vein of the dogs immediately upon arrival at the testing facility. An experienced nurse inserted the catheters in the owners and an animal caretaker inserted the catheters in the dogs.

Thirty minutes after insertion of the catheters and immediately before the owner started to interact with her dog the first blood sample (representing basal levels) was collected. The following samples were collected at 1, 3, 5, 15, 30, and 60 min after start of interaction. All blood samples were collected by an experienced nurse and an animal caretaker, respectively. They were both present in the room during the entire experiment but were instructed to ignore the participants except during the blood sampling. When not performing blood sampling they were sitting in chairs placed in one of the corners of the room.

All blood samples were collected into EDTA tubes (4 mL) containing Trasylol^®^ (aprotinin) (Bayer AB) and they were taken simultaneously from dog and owner. The samples were immediately put on ice, centrifuged, and the plasma was collected and stored at -20°C until analysis.

Plasma levels of oxytocin in both the dogs and the owners were determined using Correlate-EIA TM Oxytocin Enzyme Immunoassay kit (sensitivity 11.7 pg/mL, Intra Assay precision 9.1% and Inter Assay precision 14.5%) (Assay designs, Inc. Ann Arbor, MI, United States). Cortisol levels were determined using DSL-10-2000 ACTIVE Cortisol Enzyme Immunoassay kit (sensitivity 2.76 nmol/L, Intra Assay precision 10.3% and Inter Assay precision 8.0%) (Diagnostic Systems Laboratories, Inc., Webster, TX, United States). The procedures were performed according to the manufacturers’ instructions and the recommended standards and controls were always included. Extraction of blood samples prior to oxytocin analysis was not performed instead the samples were diluted five times in the assay buffer before analysis. The samples from the dogs were diluted two times in the zero standard buffer before analysis of cortisol levels.

An Anthos Fluido microplate washer (Anthos Labtec Instruments GmbH) was used for all washing procedures, a Multiskan Ex microplate photometer (Thermo Electron Corporation) was used for reading the absorbance. The color development was read at 405 nm with background correction at 580 nm for oxytocin, and at 450 nm with background correction at 620 nm for cortisol. Creation of standard curves, curve fitting, and calculation of concentrations was done by using Ascent software (Ascent software ver. 2.6 for iEMS Reader MF and multiskan).

One dog was excluded from the analysis of oxytocin levels, since oxtocin levels in all samples were below the range of detection for this dog.

### Video Recordings and Analysis

The entire experiment (60 min) was videotaped and the dogs’ behaviors were analyzed from the tapes. The ethogram is presented in **Table 1**. The different ways in which the owner touched her dog (stroking, scratching, or patting), and the frequency of the different types of touch as well as the total number of touching were noted. In addition the quality of the owner’s verbal interaction with her dog (rewarding or reprimanding) and the frequency of these interactions were noted, as well as the total number of verbal instructions given. The dog’s positions (sitting, standing, or lying down), and how long time they spent in each position were also noted. The frequency of the dogs’ position changes was also measured and used as an index for stress. The behavioral analysis was divided into two parts, the interaction part, i.e., 0–3 min and remaining part, i.e., 4–60 min. The frequencies of the interactive behaviors studied are summarized in **Table 2**.

**Table 1:** Ethogram describing the behaviors observed in the present study.

Behavior	Definition	Sampling method
Owner stroking	Owner strokes the dog using her palm	Continuous^a^
Owner scratching	Owner scratches the dog	Continuous^a^
Owner patting	Owner pats the dog	Continuous^a^
Owner touching	The owners’ total amount of touch (Stroking + scratching + patting)	Continuous^a^
Activating touch	The total amount of scratching and patting	Continuous^a^
Verbal rewarding	Owner reward the dog verbally (e.g., “good dog”)	Continuous^a^
Verbal reprimanding	Owner reprimands the dog verbally (e.g., “here”)	Continuous^a^
Verbal instructions	Owner give the dog verbal instructions (e.g., “sit”)	Continuous^a^
Dog sitting	Dog is sitting with front legs extended and hind legs curved	Continuous^b^
Dog standing	Dog is standing up on all four paws	Continuous^b^
Dog lying down	Dog is lying down	Continuous^b^
Dog changing position	Dog changing position (changing from sitting, lying down, and standing)	Continuous^a^

*^a^Continuous sampling by registering frequency. ^b^Continuous sampling by registering duration.*

**Table 2:** The frequency of the interaction behaviors studied.

	Total touch (number of times/time period)	Stroking (number of times/time period)	Petting (number of times/time period)	Scratching (number of times/time period)	Activating touch^∗^ (number of times/time period)
0–3 min	170 (146–206)	84 (26–111)	22 (5–55)	88 (47–141)	94 (34-150)
4–60 min	6 (1–11.5)	–	–	–	–

*Data is presented as median and quartiles (Q25-Q75). ^∗^Activating touch, the total amount of scratching and patting (**Table 1**). -, the behavior was not displayed.*

### Ethics Statement

Before start of the experiment, the owners were once more informed about the study. They were then given the opportunity to ask questions and were informed that they could end their participation in the study at any time. Written consent was obtained from all subjects in accordance with the Declaration of Helsinki.

The protocol was approved by the Local Ethics Board in Uppsala (ref. number. 2005/377).

This study was carried out in accordance with the recommendations of the Swedish Board of Agriculture. The protocol was approved by the Animal Ethics Committee in Uppsala (ref. number. 296-2005). The National Board of Agriculture approved the use of privately owned dogs.

### Statistical Analysis

The Statistical Package for the Social Sciences (SPSS, version 22.0, IBM software) was used for performing statistical calculations.

The data was not normally distributed and hence the Spearman rank coefficient was used for calculating correlations between hormone levels and behavioral data for both dogs and owners. *p*-values < 0.05 were considered significant.

The included oxytocin variables for both dogs and owners were: basal oxytocin levels, maximum oxytocin levels recorded at 1, 3, or 5 min and the delta value between basal and maximum oxytocin levels, as a measure of the increase in oxytocin levels. Cortisol variables included for both dogs and owners were: basal cortisol levels and cortisol levels at 15 min.

The following behavioral variables were included in the statistical analysis: the frequency of the owners’ stroking, scratching, patting, the total number of times the owner touched her dog, the frequency of rewarding or reprimanding verbal interaction, the total number of verbal instructions given by the owner, the time the dog spent sitting, standing, or lying down and the frequency of the dogs’ position changes. In addition, the frequency of scratching and patting were combined into one new variable, “activating touch,” which was also included in the statistical analysis (**Table 1**).

## Results

The dogs’ and the owners’ oxytocin and cortisol levels which were used in the present study have been published previously (Handlin et al., 2011) but are summarized in **Table 3**.

**Table 3:** The owners’ and the dogs’ oxytocin and cortisol levels during the experiment (data from the 10 female owners for both oxytocin and cortisol, and from nine dogs for oxytocin and ten dogs for cortsiol).

		0 min	1 min	3 min	5 min	15 min	30 min	60 min	OT max
Oxytocin levels (pmol/l)	Dogs	155.8 (26.9)	211.2 (30.7)	236.9 (38.7)	178.6 (29.6)	163.5 (34.5)	157.5 (36.0)	157.5 (41.1)	251.8 (34.5)
	Owners	168.5 (34.6)	169.8 (34.1)	180.6 (34.4)	170.2 (27.8)	146.4 (34.7)	171.3 (34.2)	165.1 (26.3)	187.0 (33.6)
Cortisol levels (nmol/l)	Dogs	168.4 (14.8)	169.4 (16.1)	168.1 (15.3)	180.1 (17.8)	224.1 (32.5)	202.8 (18.3)	190.2 (18.8)	
	Owners	389.8 (119.7)	382.7 (107.4)	382.7 (109.9)	387.6 (119.6)	362.1 (107.9)	331.6 (80.1)	305.2 (62.6)	

*Means and *SE* values (in brackets) are shown. These data have been published previously in Handlin et al. (2011).*

### Is the Frequency and Type of Touch Initiated by the Owner Associated with Oxytocin Levels in Owners and Dogs?

### The Owners

None of the different types of touch affected the owners’ oxytocin levels in specific ways but there were significant negative correlations between the owners’ basal and maximum oxytocin levels and the total number of times they touched their dogs during the 3 min of interaction; that is, owners with low oxytocin levels before and during the interaction, touched their dogs more frequently (0 min: *R*_s_ = -0.683, *p* = 0.042; oxytocin maximum value 1–5 min: *R*_s_ = -0.783, *p* = 0.013) (**Table 4**). However, the higher their oxytocin levels became during the interaction, i.e., the more the oxytocin levels increased, the less time they touched their dog during the 4th and 60th minutes (following the interaction) of the experiment (R_s_ = -0.820, *p* = 0.046) (**Table 4**).

**Table 4:** Correlation table of hormone levels and behaviors.

Hormone	Behavior	*R*_s_	*p*-value
Dog oxytocin max	Frequency of stroking 0–3 min	*R*_s_ = -0.775	*p* = 0.041^∗^
Dog cortisol 0 min	Frequency of activating touch 0–3 min	*R*_s_ = 0.648	*p* = 0.043^∗^
Dog cortisol 15 min	Frequency of activating touch 0–3 min	*R*_s_ = 0.661	*p* = 0.038^∗^
Owner oxytocin 0 min	Frequency of total touch 0–3 min	*R*_s_ = -0.683	*p* = 0.042^∗^
Owner oxytocin max	Frequency of total touch 0–3 min	*R*_s_ = -0.783	*p* = 0.013^∗^
Owner oxytocin increase	Frequency of total touch 4–60 min	*R*_s_ = -0.820	*p* = 0.046^∗^
Owner cortisol 0 min	Time dog standing up 4–60 min	*R*_s_ = 0.683	*p* = 0.041^∗^
Owner oxytocin max	Time dog sitting down 4–60 min	*R*_s_ = -0.786	*p* = 0.036^∗^
Owner oxytocin max	Frequency of the dogs’ position changes	*R*_s_ = -0.817	*p* = 0.007^∗∗^
Owner oxytocin increase	Frequency of verbal reprimands	*R*_s_ = -0.851	*p* = 0.004^∗∗^

*^∗^*p* < 0.05, ^∗∗^*p* < 0.01.*

#### The Dogs

There was a significant negative correlation between the dogs’ maximum oxytocin levels and how many times the owners stroked their dogs; that is, the lower the dogs’ oxytocin levels during the interaction, the more stroking they received (*R*_s_ = -0.775, *p* = 0.041) (**Table 4**). Besides stroking there were no significant relationships between the other forms of touch studied and the dogs’ oxytocin levels.

### Is the Frequency and Type of Touch Initiated by the Owner Associated with Cortisol Levels in Owners and Dogs?

#### The Owners

There were no significant relationships between the frequency and type of touch initiated by the owners and their cortisol levels.

#### The Dogs

There were several significant positive correlations between the frequency of activating touch (scratching and patting) during the first 3 min of the experiment and the dogs’ cortisol levels at start of the experiment but also during the remaining part of the experiment; that is, the higher the dogs’ cortisol levels were at start of interaction the more activating touch they received and the higher their cortisol levels became (0 min: *R*_s_ = 0.648, *p* = 0.043; 15 min: *R*_s_ = 0.661, *p* = 0.038) (**Table 4**). Besides for activating touch there were no significant relationships between the other forms of touch studied and the dogs’ cortisol levels.

### Are the Owners’ Oxytocin and Cortisol Levels Associated with the Dogs’ Behavior?

The owners’ maximum oxytocin levels correlated negatively to the number of position changes the dog performed during the entire experiment (R_s_ = -0.817, *p* = 0.007) and with the time the dogs were sitting between the 4th and 60th minutes (following the interaction) of the experiment (*R*_s_ = -0.786, *p* = 0.036) (**Table 4**); that is, the higher the owners’ maximum oxytocin level, the fewer position changes the dogs made during the experiment and the shorter time they spent in a sitting position.

In addition, the higher the owners’ increase in oxytocin was during the interaction, the less verbal reprimands they gave the dogs during the experiment (*R*_s_ = -0.851, *p* = 0.004) (**Table 4**).

There was a positive correlation between the owners’ basal cortisol levels and the time the dogs spent standing up during the 4th and 60th minutes (following the interaction) of the experiment; that is, the higher the owners’ cortisol in the beginning of the experiment, the longer time the dogs were in a standing position (0 min: *R*_s_ = 0.683, *p* = 0.041) (**Table 4**).

## Discussion

Based on previous data from the experiment presented in this manuscript, we have shown that interaction between owners and their dogs results in increasing levels of oxytocin in both owners and dogs, whereas cortisol levels decrease in the owners, but increase in the dogs (Handlin et al., 2011). In addition, the owners’ and the dogs’ oxytocin levels are closely related (Handlin et al., 2012). We have also shown that high oxytocin levels in both owners and dogs, and low levels of cortisol in the owners, are associated with the owner’s description of the owner-dog relationship as being pleasant, interactive and associated with fewer problems (Handlin et al., 2012).

Based on these previous results, findings from other studies, and the fact that behavioral data was also recorded in our study, we were interested in investigating whether the previously obtained oxytocin and cortisol levels in the owners and the dogs were associated with their behaviors during the interaction.

The first question addressed was whether the frequency and type of touch initiated by the owner were associated with oxytocin levels in owners and dogs. The results indicate that this is true for the owners and probably also for the dogs. Owners with lower oxytocin levels touched their dogs more frequently and dogs with lower oxytocin levels received more stroking. Since we know from previous results that the oxytocin levels in the dogs and their owners are closely related (Handlin et al., 2012) it is very likely that it was the dog–owner dyads with the lowest oxytocin levels who engaged in the most frequent interactions. It can be speculated that owners with lower oxytocin levels have a stronger need of interaction to increase their oxytocin levels and generate oxytocin mediated effects, whereas owners with higher oxytocin levels already experience oxytocin mediated effects and do not have the same need of physical interaction and hence do not interact as frequently. This interpretation is supported by the fact that the higher their oxytocin levels became during the interaction, i.e., the more the oxytocin levels increased, the less time the owners touched their dog during the 4th and 60th minutes (following the interaction) of the experiment.

The second question addressed was whether the frequency and types of touch initiated by the owner were associated with cortisol levels in owners and dogs. As presented previously in Handlin et al. (2011) the interaction decreased the owners’ cortisol levels. Since oxytocin is known to inhibit cortisol release (Neumann et al., 2000), this decrease in cortisol levels is probably a result of the increased oxytocin levels as a consequence of the tactile interaction. The results from the present analysis did, however, not show any significant relationships between the frequency and types of touch and cortisol levels in the owner.

In contrast, the dogs’ cortisol levels correlated positively and significantly with the amount of activating touch they received from their owners. In everyday-life interactions between dogs and owners, the activating type of touch is probably used more frequently during play. On the other hand, the stroking type of touch is probably used more frequently during calm interaction between owners and their dogs and hence it might have a more calming effect on the dogs. The activating touch applied to the dogs in this study might therefore have triggered an expectation of play in the dogs.

It is important to point out that the observed increase in the dogs’ cortisol levels does not appear to have anything to do with stress, since they did not display behaviors related to stress (looking at frequency of position changes). The increasing cortisol levels are therefore probably a reflection of positive arousal (Lewandowski et al., 2014) and preparation for and expectation of activity in the dogs (Horváth et al., 2008).

The third question addressed was whether the owners’ oxytocin and cortisol levels were associated with the dogs’ behavior. According to the results from the present analysis it appears as if there are associations between the owners’ oxytocin levels and the dogs displaying calm behaviors. This was demonstrated by the findings showing that the higher the owners’ maximum oxytocin level, the fewer position changes the dogs made during the experiment and the shorter time they spent in a sitting position. Together these data suggest that high oxytocin levels in the owners are associated with a friendly and calm behavior toward the dog and hence with calming effects in the dogs.

It is also possible that the owners with high cortisol displayed a more active, or even stressed, behavior which influenced the dogs, as demonstrated by the finding showing that the higher the owners’ cortisol were in the beginning of the experiment, the longer time the dogs spent in a standing position.

It appeared as if the dogs and their owners responded to the interaction in similar ways with regard to oxytocin. The interaction induced oxytocin release in the owners who displayed behaviors that are associated with anti-stress effects. The dogs seemed to sense this and responded in a similar way. The calmer behaviors displayed by the dog then enhanced the calming effect in the owners. It appears as if the owners and the dogs could mutually sense the other’s emotional state based on an increased ability to read the other’s behavioral cues. As previously described oxytocin can facilitate and stimulate friendly social interactions, induce anti-stress and anxiolytic effects and increase trust. It has been shown that oxytocin relates to the level of maternal interaction and sensitivity to the infant cues (Feldman et al., 2007), but also to more frequent interaction between dog owners and dogs (Handlin et al., 2011, 2012).

The results from the present and previous studies suggests that the activity and effect of the oxytocinergic system are probably part of a “mammalian heritage” that can be activated by individuals from different species, as in the interspecies relationship seen between dogs and their owners, and not only by individuals from the same species. Due to their evolutionary history (Miklósi, 2009), dogs and humans have been suggested to be especially good at activating each other’s oxytocinergic systems and generating oxytocin-linked effects (Beetz et al., 2012).

Even though both dogs and owners responded in similar ways with regard to oxytocin some differences were noticed in the responses related to cortisol. An explanation to the mismatch in cortisol responses between the owners and the dogs might be that the interaction was driven by the owners, rather than a reciprocal, two-way decision process. The owners knew that it was going to be a calm interaction and were prepared for this and initiated the contact. The dogs on the other hand were unaware of what type of interaction that was going to take place and since the interaction was driven by the owners it might have caused some confusion for the dogs. This could explain the difference in responses between dogs and their owners.

To keep variations due to breed and gender to a minimum we chose to study male Labrador dogs and their middle-aged female owners. Labradors are friendly and easy to work with and is one of the most common types of companion dogs. However, in future studies it would be interesting to study both female and male dogs and dog owners but also other breeds.

We are aware that the number of participants in this study is low (10 dog–owner pairs) and that the results need to be interpreted with caution but still the results may serve as proof of a concept. One of the strengths of the study is the study design which included repeated sampling. This made it possible to detect the interplay between oxytocin concentrations and physical contacts. Studies with a larger number of participants and performed under even more standardized conditions need to be done to help gaining a better understanding of the responses in dogs and their owners as a consequence of interaction.

## Conclusion

The present study showed that oxytocin and cortisol levels, in both dogs and their owners, are associated with the way the owners interact with their dogs and also with behaviors caused by the interaction.

## Author Contributions

MP: involved in study design and writing manuscript. KU-M: main applicant for funding, responsible for study design, involved in writing manuscript. AN: involved in data collection and writing manuscript. L-LG: involved in data analysis and writing manuscript. EH-S: involved in funding, study design and writing manuscript. LH: involved in study design, main responsible for data collection, statistical analysis and writing manuscript.

## Conflict of Interest Statement

The authors declare that the research was conducted in the absence of any commercial or financial relationships that could be construed as a potential conflict of interest.
